# CYT387, a potent IKBKE inhibitor, suppresses human glioblastoma progression by activating the Hippo pathway

**DOI:** 10.1186/s12967-021-03070-3

**Published:** 2021-09-20

**Authors:** Xin Wang, Jie Lu, Jing Li, Yang Liu, Gaochao Guo, Qiang Huang

**Affiliations:** 1 Department of Radiation Oncology, Shandong Cancer Hospital and Institute, Shandong First Medical University and Shandong Academy of Medical Sciences, Jinan, Shandong China; 2 Department of Neurosurgery, The First Affiliated Hospital of Shandong First Medical University & Shandong Provincial Qianfoshan Hospital, Shandong Medicine and Health Key Laboratory of Neurosurgery, Jinan, Shandong China; 3 Department of Nursing, The First Affiliated Hospital of Shandong First Medical University & Shandong Provincial Qianfoshan Hospital, Jinan, Shandong China; 4 Department of Neurosurgery, Renmin Hospital of Henan Province, Zhengzhou, Henan China; 5 Department of Neurosurgery, Tianjin Medical University General Hospital, Tianjin, China; 6 Tianjin Neurological Institute, Key Laboratory of Post-Neuroinjury Neuro-repair and Regeneration in Central Nervous System, Ministry of Education and Tianjin City, Tianjin, People’s Republic of China

**Keywords:** CYT387, IKBKE, Glioblastoma, Hippo pathway

## Abstract

Recent studies have showed that IKBKE is overexpressed in several kinds of cancers and that IKBKE-knockdown inhibits tumor progression. In this article, we first verified that two glioblastoma cell lines, U87-MG and LN-229, were sensitive to CYT387 by measuring the half maximal inhibitory concentration (IC50) with a CCK-8 assay and then demonstrated that CYT387, as a potent IKBKE inhibitor, suppressed glioblastoma cell proliferation, migration and invasion. Additionally, CYT387 induced cell apoptosis and arrested the cell cycle at the G2/M checkpoint in vitro. Furthermore, we showed that CYT387 did not simply inhibit IKBKE activity but also decreased IKBKE expression at the protein level rather than at the mRNA level. We discovered that CYT387 restrained malignant tumor progression by activating the Hippo pathway in vitro. By coimmunoprecipitation (co-IP), we showed that IKBKE interacted with TEAD2 and YAP1, thus accelerating TEAD2 and YAP1 transport into the nucleus. In subsequent in vivo experiments, we found that CYT387 inhibited subcutaneous nude mouse tumor growth but had little impact on intracranial orthotopic xenografts, probably due to a limited ability to penetrate the blood–brain barrier (BBB). These results suggest that CYT387 has potential as a new antiglioblastoma drug, but an approach to allow passage through the blood–brain barrier (BBB) is needed.

**Supplementary Information:**

The online version contains supplementary material available at 10.1186/s12967-021-03070-3.

## Introduction

Glioblastoma, a common intracranial primary malignant glioma, has high recurrence, morbidity and mortality rates. Although the most effective therapy includes surgical resection, radiotherapy with concomitant and adjuvant temozolomide has been widely adopted, however, the poor patient prognosis, which is represented by the 5-year survival rate of 9.8% and median survival time of 14.6 months, remains to be improved [1, 2]. Due to the high heterogeneity, low immunogenicity and immunosuppressive microenvironment, the effective immune checkpoint inhibitor therapy and tumor target therapy for many tumors have little benefit in glioblastoma [3, 4]. Glioblastoma has become an intractable disease in neurosurgery, emphasizing that there is an urgent need to delineate the underlying molecular mechanisms and identify new treatment strategies.

IKBKE, also called IKKε or IKKi, belongs to the IκB kinase (IKK) family that induces noncanonical NF-κB signaling [5, 6]. In prior studies, IKBKE has been demonstrated to be a novel oncogene in breast cancer and was shown to be amplified in over 30% of breast cancer cases [7]. Recent studies have also shown that IKBKE is overexpressed in glioma [8–10], ovarian cancer [10], prostate cancer [11, 12], non-small cell lung cancer [13, 14], gastric cancer [15] and renal clear cell carcinoma [16]. These studies have revealed that IKBKE has close relationships with cancer pathological grade or clinical stage in glioma [9], ovarian cancer [10], and lung squamous cell cancer [14, 17] and induces tumor chemoresistance in ovarian cancer [11] and non-small cell lung cancer [13]. In addition, overexpression of IKBKE results in malignant cell transformation [7, 14]. Li et al. [9] showed that silencing IKBKE inhibited glioma proliferation in vitro and in vivo, indicating that IKBKE can contribute to glioma progression. Taken together, these data indicate the importance of IKBKE in tumorigenesis and suggest that inhibiting IKBKE expression may represent a new approach for the treatment of malignancy [18].

CYT387 (also named momelotinib), traditionally considered a JAK1 and JAK2 inhibitor, has been used in the clinical treatment of myelofibrosis. However, an increasing number of extensive studies have focused on its usage in myeloproliferative neoplasms [19] and other tumors. Lue et al. [20] reported that the combined use of CYT387 (a JAK/STAT inhibitor) and dasatinib (an Src inhibitor) synergistically reduced cell proliferation and increased apoptosis in renal cell carcinoma. Hu et al. [21] pointed out that CYT387 in combination with cetuximab (an EGFR inhibitor) effectively inhibited non-small cell lung cancer proliferation, especially that of EGFR inhibitor-resistant tumors. Zhu et al. [22] revealed that CYT387 could suppress IKBKE kinase activity in an in vitro kinase assay, thus blocking KRAS-dependent lung cancer cell growth. Barbie et al. [23] showed that CYT387, as a TBK1/IKBKE/JAK inhibitor, inhibited triple-negative breast cancer proliferation by suppressing the NF-κB and STAT3 activation induced by IKBKE; however, there was almost no inhibitory effect on cancer growth if only JAK activity was suppressed. Generally, CYT387 can inhibit both IKBKE activation and JAK/STAT activation to regulate tumor growth via a complicated pathway. However, the specific function of CYT387 in glioblastoma remains to be elucidated.

The Hippo pathway regulates cell proliferation, differentiation, tissue development and stemness in conjunction with inputs from the intracellular and extracellular microenvironments, including cell contact, cell polarity, and mechanotransduction [24, 25]. Dysregulation of the Hippo pathway can cause cancer development. In recent studies, Ji et al. [26] pointed out that the LATS1 expression level was remarkably decreased in glioma tissues and had a close relationship with the tumor grade and prognosis of patients. Orr et al. [27] showed that YAP1 was overexpressed in high-grade glioma and that knocking down YAP1 expression obviously inhibited glioma cell line proliferation, suggesting that YAP1 plays an important role in glioma progression. These data indicate that inactivation of the Hippo pathway leads to glioblastoma progression and that the Hippo pathway is a candidate for therapeutic manipulation.

In this article, we first demonstrated that CYT387, as an IKBKE inhibitor, inhibited glioma cell proliferation, migration, and invasion in vitro; accelerated cell apoptosis; and arrested the cell cycle at the G2/M checkpoint. In addition, we verified that CYT387 increased Hippo pathway activity to inhibit glioma malignancy and that IKBKE directly interacted with YAP1 and TEAD2, as determined by using coimmunoprecipitation (co-IP). Additionally, inhibition of IKBKE suppressed YAP1 and TEAD2 translocation into the nucleus. Moreover, we showed that CYT387 could suppress glioblastoma growth in a subcutaneous nude mouse model but had little impact on intracranial orthotopically implanted tumors. Our data indicate that CYT387 may become a new anticancer drug of interest for glioblastoma treatment, but its limited ability to penetrate the blood–brain barrier (BBB) needs to be addressed.

## Materials and methods

### Cell culture, transfection and antibodies

The human glioblastoma cell lines U251 and LN229 came from the Institute of Biochemistry and Cell Biology (Shanghai, China). HEK293 cells were from the Institute of Biochemistry and Cell Biology (Shanghai, China). All cells were maintained in Dulbecco's modified Eagle's medium (DMEM, Gibco, USA) supplemented with 10% fetal bovine serum (Gibco, USA), and cultured at 37 °C in 5% CO_2_. We established an IKBKE-shRNA lentiviral vector from GeneChem (Shanghai, China) with the sequence of 5ʹ-GCATCATCGAACGGCTAAATA-3ʹ. A GFP scrambled lentiviral vector with the sequence of 5ʹ-TTCTCCGAACGTGTCACGTTTC-3ʹ was used as the negative control. The shRNAs were transfected according to the manufacturer’s instructions. The Flag-IKBKE plasmid was purchased from Addgene (USA). The HA-TEAD2 and HA-YAP1 plasmids were from Hanbio Biotechnology (Shanghai, China). The IKBKE-overexpress lentivirus and its empty vector virus were from GeneChem (Shanghai, China). We selected Lipofectamine 3000 (thermo fisher scientific, USA) as transfection medium and the process of transfection was according to the manufacturer’s instructions. IKBKE rabbit mAb (No.2905,WB 1:1000; IP 1:100), c-myc rabbit mAb (No.13987,WB 1:1000), MMP9 rabbit mAb (No.13667,WB 1:1000), YAP1 mouse mAb (No.12395,WB 1:1000; IHC 1:400; IP 1:100), Bcl-2 rabbit mAb (No.2870,WB 1:1000), Phospho-YAP (Ser127) rabbit mAb (No.13008,WB 1:1000; IHC 1:2000), HA-Tag rabbit mAb (No.3724,WB 1:1000; IP 1:50), DYKDDDDK-Tag (Flag) rabbit mAb (No.14793,WB 1:1000; IP 1:50) and Axl rabbit mAb (No.8661,WB 1:1000;IHC 1:300) were purchased from Cell Signaling Technology (USA). IKBKE rabbit polyclonal antibody (ab7891,IHC 1:100), Cdk1 rabbit mAb (ab133327,WB 1:10000;IHC 1:300), Cdc25c rabbit mAb (ab32444,WB 1:2000), caspase-9 rabbit mAb (ab202068,WB 1:2000), Bax rabbit mAb (ab32503,WB 1:2000), CyclinB_1_ rabbit mAb (ab32053,WB 1:5000;IHC 1:100), CyclinA_2_ rabbit mAb (ab181591,WB 1:2000), c-myc rabbit mAb (ab32072,IHC 1:500), CyclinD_1_ rabbit mAb (ab134175,WB:1:10000), TEAD2 rabbit polyclonal antibody (ab83670, WB 1:500) were purchased from Abcam (USA). TEAD2 rabbit polyclonal antibody (sc-67115, IP 1:50) was from Santa Cruz (USA). LATS2 rabbit polyclonal antibody (20276-1-AP, WB 1:500; IHC 1:50) were purchased from proteintech (USA). GAPDH mouse mAb (WB 1:2000) was from ZSGB-Bio (Beijing, China).

### Protein extraction and western blot analysis

The cell total protein was extracted after treatment of CYT387, plasmids or IKBKE-shRNA using RIPA lysis buffer with protease and phosphatase inhibitor (MCE USA). The homogenates were clarified by centrifugation at 4 °C for 15 min at 12,000 rpm after cleavage by RIPA for 15 min, and the protein concentration was measured by BCA assay kit (Beyotime, Shanghai, China). At least 20 μg protein mixed with 4 × loading buffer was added into spacer gel and then separated by sodium dodecylsulphate-polyacrylamide gel electrophoresis (SDS-PAGE). The protein bands were electrotransferred to PVDF membranes (Millipore, USA). Primary antibodies were incubated at 4 °C for overnight then HRP-conjugated secondary antibody (1:3000 dilution, ZSGB-Bio, Beijing, China) was used for 1 h at room temperature. The bands were detected by the G:BOX (Syngene Company, UK) using Chemiluminescent HRP Substrate (Millipore USA).

### RNA extraction and real-time RT-PCR analysis

Total RNA of glioblastoma cells (U87-MG and LN-229) after treatment with CYT387 in dose- and time-dependent manners was extracted by TRIzol reagent (Invitrogen, USA) following the manufacturer’s protocols and then reverse transcription was performed using GoScript™ Reverse Transcription System (Promega, USA). The quantitative real-time PCR was finished by GoTaq qPCR Master Mix (Promega, USA) according to the supplier’s instructions. The reaction conditions were as follows: 95 °C for 5 min and 40 cycles of 95 °C for 12 s and 60 °C for 40 s. The primers were synthesized by GENEWIZ (USA). The sequences of the primers were as follows: GAPDH: 5ʹ-GGAGCGAGATCCCTCCAAAAT-3ʹ (Forward primer) and 5ʹ-GGCTGTTGTCATACTTCTCATGG-3ʹ (Reverse primer); IKBKE: 5ʹ-GAGAAGTTCGTCTCGGTCTATGG-3ʹ (Forward primer) and 5ʹ-TGCATGGTACAAGGTCACTCC-3ʹ (Reverse primer); TEAD2: 5ʹ-GCCTCCGAGAGCTATATGATCG-3ʹ (Forward primer) and 5ʹ-TCACTCCGTAGAAGCCACCA-3ʹ (Reverse primer); YAP1: 5ʹ-TAGCCCTGCGTAGCCAGTTA-3ʹ (Forward primer) and 5ʹ-TCATGCTTAGTCCACTGTCTGT-3ʹ (Reverse primer). GAPDH was used as internal control.

### Clone formation assay

U87-MG and LN-229 cells were seed in six-well plates (2 × 10^3^/well) divided into three groups as blank control, negative control (DMSO) and drug group (CYT387 with concentration of 6 μM). Growth medium was changed every 6 days. After 12 days, cells were fixed in 4% paraformaldehyde for 15 min and stained with crystal violet for 30 min. Colonies were scored after photographed.

### CCK-8 assay

For IC_50_ measurement, we seeded U87-MG and LN-229 cells (5000/50 ul/well) into 96-well plates on the first day. After cells were adherent, we again added 50ul/well medium with different concentration of CYT387 to achieve the final drug concentration gradient of 0.5 μM, 1 μM, 2 μM, 4 μM, 8 μM, 16 μM, 32 μM and 64 μM. After treatment with CYT387 for 24, 48, and 72 h, 10 μl CCK8 reagent (dojindo, Japan) was mixed into each well and then 96-well plate was incubated for 2 h at 37˚C. The O.D. value was measured by Microplate reader at the wavelength of 450 nm.

For proliferative curve measurement, glioblastoma cells with normal medium, DMSO medium and 6 μM CYT387 medium were seeded (2000/100 µl/well) into 96-well plates. From first day to fifth day, 10 µl CCK-8 reagent (dojindo, Japan) was added into each well. The O.D. value was measured after incubated for 2 h at 37 °C in a 5% CO_2_ atmosphere.

### Wound healing assays

U87-MG and LN-229 cells were seeded in six-well plate and a straight wound was created with a sterile 100 µl pipette tip. Then we respectively added DMSO and CYT387 in DMSO group and drug group, making the drug concentration of 6 μM. After treatment for 24 h and 48 h, the wound healing area was detected by an inverted microscope.

### Transwell assay

Before experiment begun, matrigel with 3 times volume serum-free DMEM (total 80 µl/well) was coated on the upper surface of chamber. Then place it at 37 °C for 30 min, waiting for matrigel solidification. Then the cells (5 × 10^4^/well) were seeded into the transwell chambers with 200 μl serum-free DMEM while outer space was filled with 500 μl serum DMEM. After incubated at 37 °C in a 5% CO_2_ atmosphere for 48 h, cells across the chamber membrane was fixed with 4% paraformaldehyde for 15 min, then stained with crystal violet for 5 min, counted and imaged under the microscope.

### Cell apoptosis assays and cell cycle analysis

Before cell apoptosis assay, the cells were treated with CYT387 with concentration of 6 μM for 24 h. Cells were trypsinized without EDTA, washed with PBS twice and then stained using the Annexin V-FITC Apoptosis Detection kit from KeyGen Biotech (Nanjing, China) according to the manufacturer’s instructions. Flow cytometry analysis was finished by a FACS flow cytometer (Becton–Dickinson). Data were analyzed by CellQuest software. Before cell cycle analysis, the U87-MG and LN-229 cells was treated with CYT387 at the concentration of 6 μM for 72 h. Then the following protocols were performed as the previous article [9].

### Co-immunoprecipitation (co-IP)

Co- immunoprecipitation (co-IP) was carried out as described previously [28].

### Animal studies

Before animal studies, CYT387 purchased from selleck (USA) was dissolved in NMP (1-methyl-2-pyrrolidinone) to finally get the concentration of 120 mg/ml. Next, the CYT387 was diluted with 0.14 M Captisol (MCE, USA) to a concentration of 6 mg/ml. The tumor subcutaneous experiments method was carried out as described previously [9]. After subcutaneous tumour was shaped, the nude mice were fed with CYT387 (100 mg/kg/day).

For intracranial orthotopic model, U87-MG transfected with luciferase-expressing lentivirus was injected intracranially into 6-week-old BALB/c-nu mice. After 7 days, mice started to be fed with CYT387 (100 mg/kg/day). The weight of mice was monitored every 2 days and the luminescence imaging of intracranial tumour was measured using an IVIS Lumina Imaging System (Xenogen) every 7 days.

### Immunohistochemical staining

After mice were sacrificed for subcutaneous and intracranial tumour, we made specimens embedded with paraffin. Then the paraffin-embedded tumours were sectioned and dewaxed. After antigen retrieval using 10 mmol/l citrate buffer, sections were incubated with 3% H_2_O_2_ and blocked with 5% BSA. Then the sections were added with primary antibodies at 4 °C overnight. After rewarming at room temperature the next day, the sections were incubated with secondary antibodies using two-step polymer HRP detection system (ZSGB-BIO, Beijing, China). The samples were colourated with DBA Kit (ZSGB-BIO, Beijing, China) and then counterstained with haematoxylin. After dehydration and sealing piece with neutral gum, the samples were detected and photographed by microscope (Olympus Japan).

### Statistical analysis

All data were repeated at least three times. Quantitative data are shown as the mean ± standard deviation (SD). We used SPSS software (version 16.0) for the statistical analyses and P < 0.05 was considered statistically significant.

## Results

### CYT387 remarkably inhibits glioblastoma cell proliferation in vitro

**Fig. 1:**
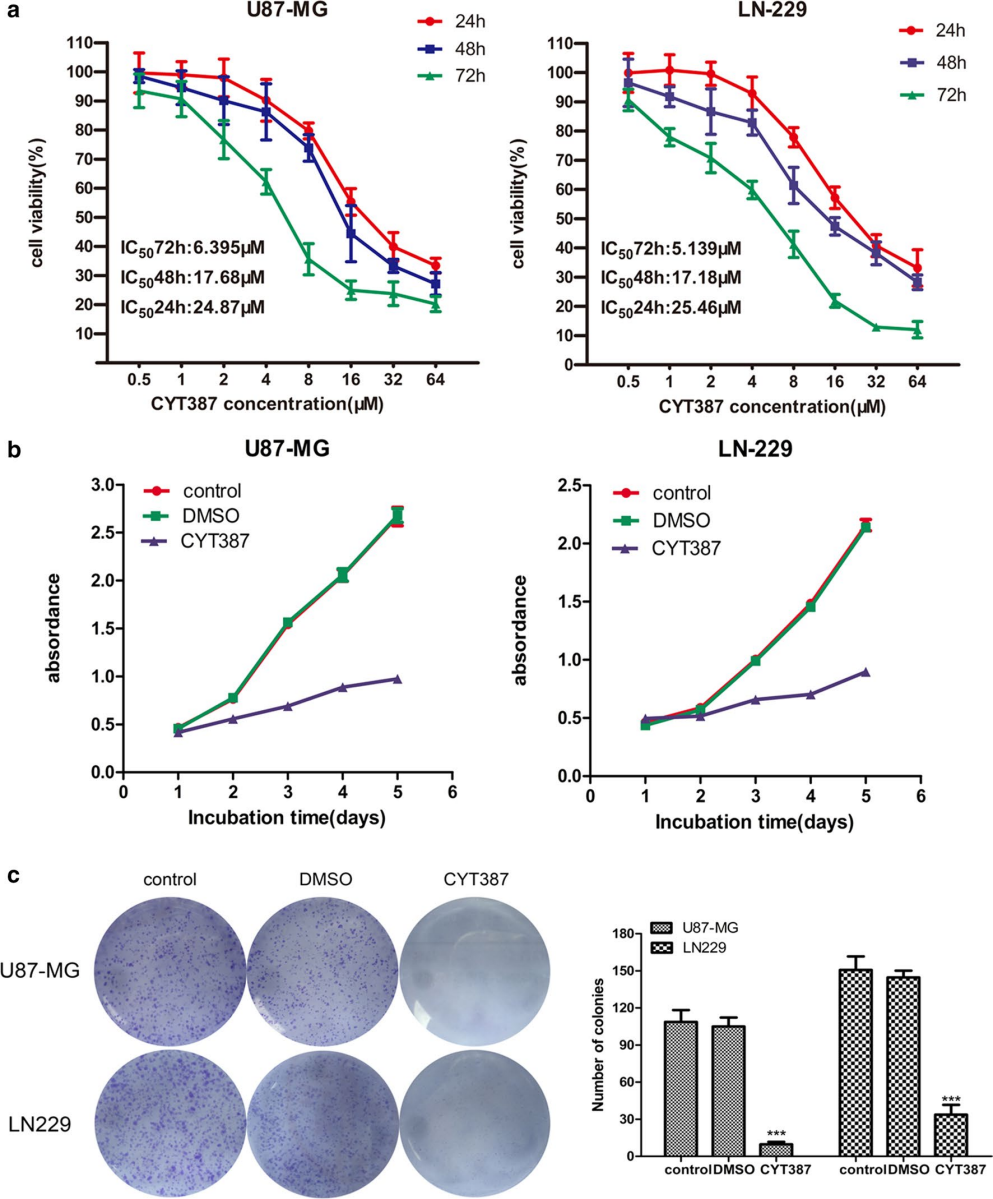
CYT387 inhibits proliferation in glioblastoma cells. **A** Half maximal inhibitory concentration (IC50) of CYT387 was measured by CCK-8 in U87-MG and LN229 cells. **B** Glioblastoma cells treated with CYT387 dramatically decreased the capacity of proliferation using CCK-8 assay. **C** Colony formation assay showed decreased ability of forming colonies after treatment with CYT387. All experiments were repeated at least three times. (*p < 0.05; **p < 0.01; ***p < 0.001)

To evaluate the sensitivity of glioblastoma cells to CYT387 in vitro, two glioblastoma cell lines that highly express IKBKE, U87-MG and LN-229 [10], were treated with a concentration gradient of CYT387 (0.5 μM to 64 μM) to measure the half maximal inhibitory concentration (IC50) with a CCK-8 assay. A dose–response curve with data points for 24 h, 48 h, and 72 h of drug treatment is shown in Fig. 1a. The IC50 values of U87-MG cells were 6.395 ± 1.127 μM for 72 h, 17.68 ± 2.94 μM for 48 h, and 24.87 ± 3.63 μM for 24 h, and those of LN229 cells were 5.139 ± 0.501 μM for 72 h, 17.18 ± 2.61 μM for 48 h, and 25.46 ± 3.59 μM for 24 h. These data demonstrated that glioblastoma cells were sensitive to CYT387, especially after 72 h of treatment. Then, we carried out a CCK-8 assay to investigate the effect of CYT387 on glioblastoma cell proliferation using a drug concentration of 5 μM and incubating the cells for 5 days. The results (Fig. 1b) showed that the viability of U87-MG and LN229 cells treated with CYT387 was dramatically decreased compared with that of the cells in the blank control and DMSO (drug solvent) groups. Additionally, a colony formation assay was used to test whether CYT387 affects the ability of the two cell lines to form colonies over a period of 12 days. Figure 1c demonstrates that compared with those in the blank control and solvent groups, the cells treated with the drug at a concentration of 6 μM had a markedly decreased number of colonies, and the colonies in the groups treated with the drug were much smaller than those in the blank control and DMSO groups (P < 0.001). These data showed that CYT387 could significantly inhibit glioblastoma cell proliferation in vitro.

### CYT387 dramatically inhibits the migration and invasion of glioblastoma cell lines in vitro

**Fig. 2:**
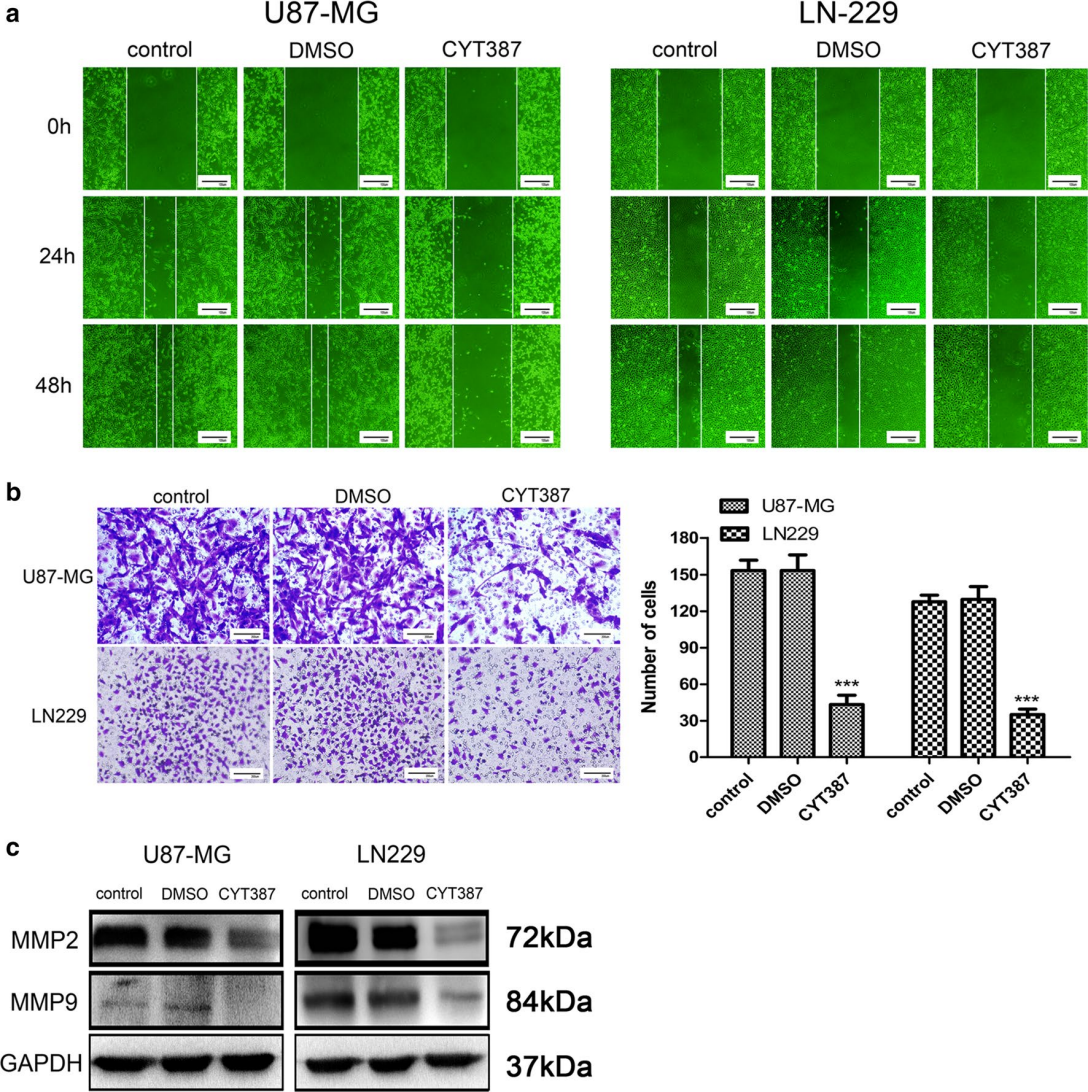
CYT387 inhibits migration and invasion in glioblastoma cells. **A** Migration ability of U87-MG and LN229 was measured by wound healing test after treated with CYT387. **B** Invasion capacity of glioblastoma cells was measured by transwell assay after treated with CYT387. **C** MMP2 and MMP9 were detected by western blot after treatment with CYT387. All experiments were repeated at least three times. (*p < 0.05; **p < 0.01; ***p < 0.001)

To explore whether CYT387 impacts tumor cell migration, a wound healing assay was adopted to assess the adherent tumor cell healing area at different times with drug treatment at a concentration of 5 μM. Compared with control or solvent treatment, CYT387 markedly inhibited the wound healing speed after only 24 h, suggesting a poor migratory ability in the CYT387 group (Fig. 2a). Furthermore, we investigated the influence of CYT387 on the glioblastoma cell invasive capacity using a Transwell assay. The experimental results (Fig. 2b) showed that the average number of invaded cells in the group treated with a drug concentration of 6 μM was significantly decreased compared to that in the blank control and solvent groups after 48 h, showing that CYT387 had an inhibitory effect on the invasive ability of U87-MG and LN229 cells. To further research the detailed mechanism, two important matrix metalloproteinases (MMPs), MMP2 and MMP9, were assessed by western blot analysis. As Fig. 2c shows, MMP2 and MMP9 expression levels were significantly reduced after tumor cells were treated with CYT387 for 48 h compared with control or DMSO treatment (Figure S2). These data fully demonstrated that CYT387 could effectively inhibit glioblastoma cell migration and invasion.

### CYT387 accelerates glioblastoma cell apoptosis

**Fig. 3:**
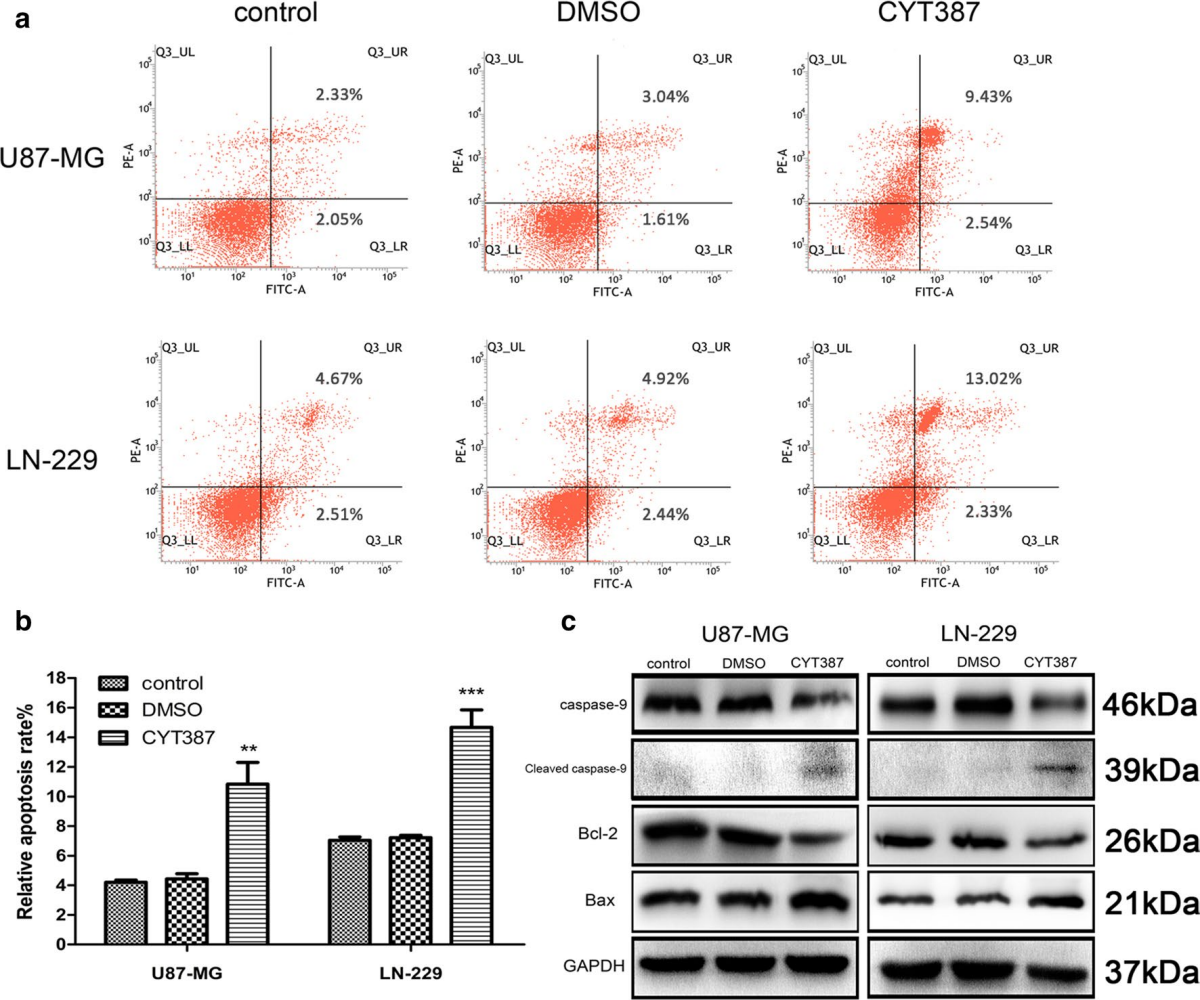
CYT387 accelerates glioblastoma cells apoptosis. **A**, **B** CYT387 accelerated U87-MG and LN229 cells apoptosis compared to DMSO and blank control groups. **C** Apoptotic markers caspase-9, cleaved caspase-9, Bcl-2, Bax were detected by western blot after treatment with CYT387. All experiments were repeated at least three times. (*p < 0.05; **p < 0.01; ***p < 0.001)

Since apoptosis is critical to tumor regression, we performed Annexin V-FITC/PI staining using flow cytometry to test whether CYT387 can influence glioblastoma cell apoptosis. After cells were incubated with the drug for 24 h at a concentration of 5 μM, the tumor cell apoptosis rate was obviously increased compared with that observed in the blank control and DMSO groups (Fig. 3a, b). The apoptosis rates of U87-MG cells were 4.20 ± 0.127% in the blank group, 4.47 ± 0.287% in the DMSO group and 10.8 ± 1.20% in the drug group (P < 0.01), while the apoptosis rates of LN229 cells were 7.04 ± 0.176% in the blank group, 7.21 ± 0.138% in the DMSO group and 14.67 ± 0.960% in the drug group (P < 0.001). Moreover, the mechanism underlying the induction of apoptosis by CYT387 was assessed by western blot analysis. The protein expression of caspase-9 was decreased, but that of cleaved caspase-9 was increased in the groups treated with 5 μM drug for 24 h compared with the blank control and DMSO groups (Fig. 3c and Figure S2). Additionally, the antiapoptotic protein Bcl-2 and proapoptotic protein Bax were assessed. As shown in Fig. 3c, Bcl-2 expression was decreased, but Bax expression was increased after incubation with the drug (6 μM) for 24 h. All data indicated that CYT387 could obviously accelerate glioblastoma cell apoptosis.

### CYT387 arrests the glioblastoma cell cycle at the G2/M checkpoint

**Fig. 4:**
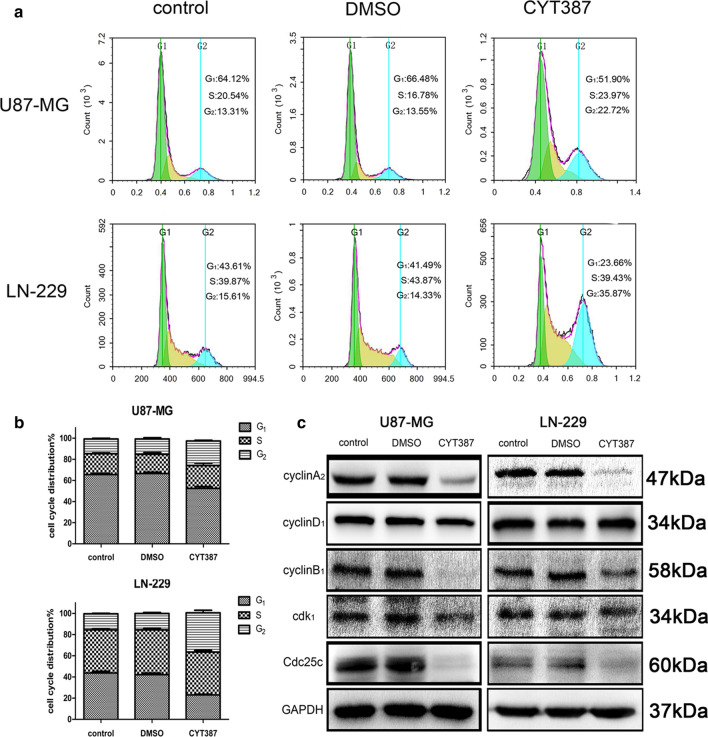
CYT387 arrests glioblastoma cell cycle on G2/M phase. **A**, **B** CYT387 arrested U87-MG and LN229 cell cycle on G2/M phase. **C** Cell cycle markers cyclinA_2_, cyclinD_1_, cyclinB_1_, cdk1 and Cdc25c were detected by western blot after treatment with CYT387. All experiments were repeated at least three times. (*p < 0.05; **p < 0.01; ***p < 0.001)

We found that treatment with CYT387 (5 μM) for 3 days induced cell cycle arrest at the G2/M checkpoint. As shown in Fig. 4a, b, the proportions of cells at the G2/M checkpoint for U87-MG cells were 23.23 ± 0.54% in the drug group, 13.90 ± 0.50% in the blank control group and 14.47 ± 1.00% in the solvent group (P < 0.001), while those for LN229 cells were 37.03 ± 1.96% in the drug group, 15.11 ± 0.39% in the blank control group and 15.44 ± 0.79% in the solvent group (P < 0.001). In addition, the proportions of glioblastoma cells in the G1 and S phases were correspondingly decreased. In addition, we examined the expression levels of several cell cycle kinases including CyclinA2, CyclinD1, CyclinB1, Cdk1 and Cdc25c by western blot analysis. As shown in Fig. 4c, the expression of CyclinD1, an important regulator driving cell transition from the G1 phase into the S phase, exhibited a negligible change, while the levels of CyclinA2, CyclinB1, Cdk1 and Cdc25c, as several essential factors regulating the mitotic entry of cells from the G2/M checkpoint, were significantly decreased in the drug group (6 μM) compared to the blank control and DMSO groups (Figure S2). All this information suggested that CYT387 induced glioblastoma cell cycle arrest at the G2/M checkpoint to inhibit cell proliferation.

### CYT387 activates the Hippo signaling pathway by inhibiting IKBKE expression to suppress glioblastoma cell growth

**Fig. 5:**
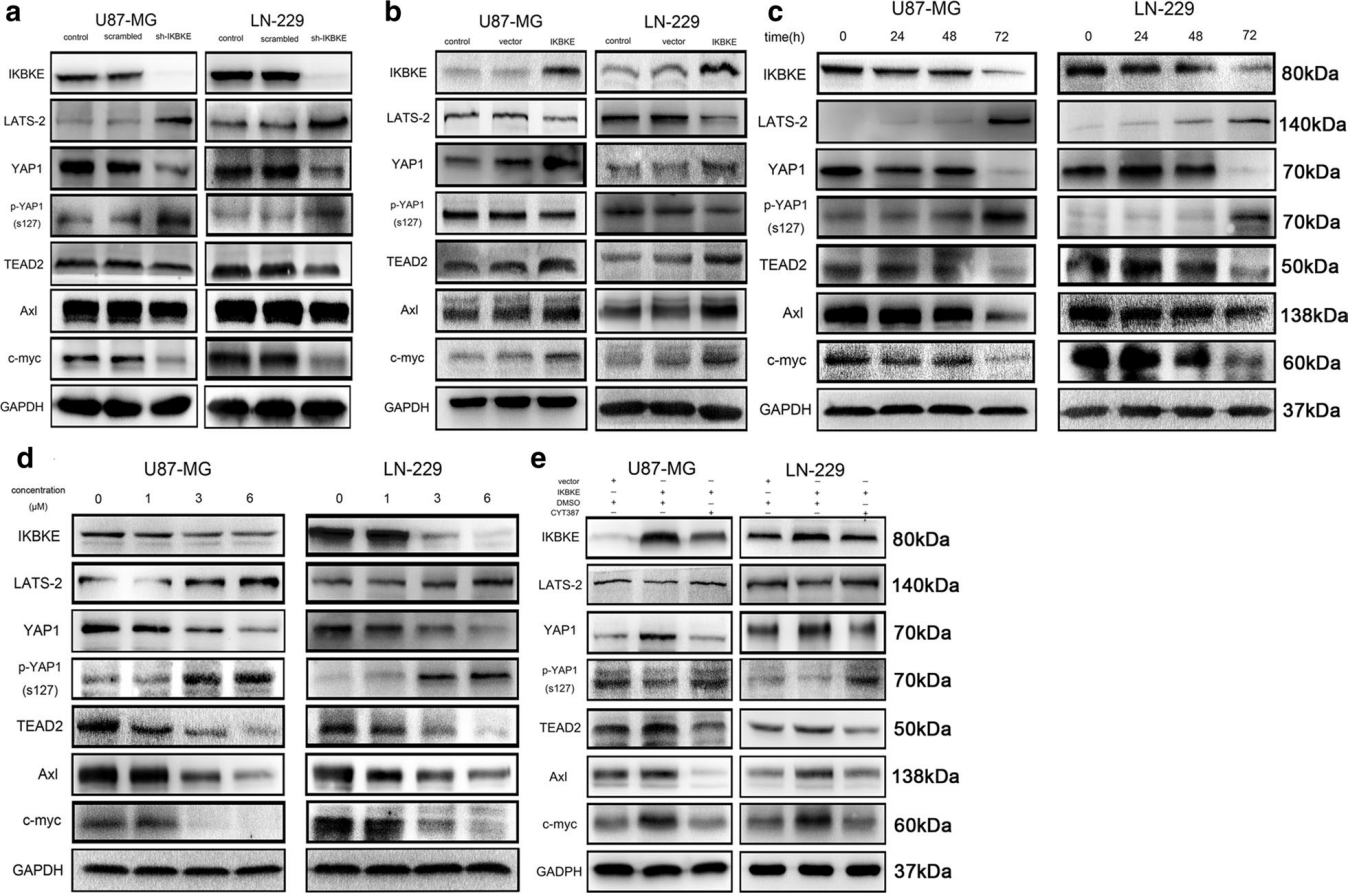
CYT387 activates the Hippo pathway via inhibiting IKBKE expression. **A** IKBKE and the Hippo pathway markers LATS-2, YAP1, p-YAP1, TEAD2 and its downstream factors Axl and c-myc were detected by western blot after knockdown of IKBKE in U87-MG and LN229. **B** IKBKE and the Hippo pathway markers LATS-2, YAP1, p-YAP1, TEAD2 and its downstream factors Axl and c-myc were detected by western blot after overexpression of IKBKE in U87-MG and LN229. **C**, **D** IKBKE, LATS-2, YAP1, p-YAP1, TEAD2, Axl and c-myc were measured by western blot after treatment with CYT387 in a dose-dependent and a time-dependent manner. **E** The expressions of IKBKE, LATS-2, YAP1, p-YAP1, TEAD2, Axl and c-myc were measured by western blot after overexpressing IKBKE and then treatment with CYT387 for 72 h. All experiments were repeated at least three times

The Hippo pathway, which is composed of a cascade of phosphorylated kinases, can regulate cell proliferation, apoptosis and differentiation. The core Hippo pathway has been well established in mammals. MST1/2 directly phosphorylates LATS1/2 with the help of Sav1 and Mob1. Then, LATS1/2 directly interacts with and phosphorylates YAP1 at Ser127, resulting in YAP sequestration in the cytoplasm and degradation via ubiquitylation by 14-3-3 [24, 25]. When YAP1 is unphosphorylated, due to an ineffective Hippo pathway, it is transported into the nucleus to interact with TEAD1-4, creating transcription factors that induce the transcription of certain genes [29, 30], such as Axl [31], c-myc [32], Cyr61 [33, 34], and CTGF [34]. We showed that the levels of the core Hippo pathway effectors YAP1 and TEAD2 and their downstream factors including Axl or c-myc were decreased, while those of LATS2 and p-YAP1 (S127) were increased in the IKBKE-knockdown group compared with the blank control and scrambled groups by western blot analysis (Fig. 5a and Figure S3), indicating that IKBKE inhibition enhanced Hippo pathway activity by decreasing YAP1 and TEAD2 expression and increasing the expression of LATS2, which could accelerate the phosphorylation and degradation of YAP1. Additionally, overexpression of IKBKE increased YAP1 and TEAD2 expression, as well as the expression of the downstream factors Axl and c-myc, while reducing LATS2 and p-YAP1 (S127) expression (Fig. 5b and Figure S3). Furthermore, the effect of CYT387 on the Hippo pathway was confirmed to be mediated in a dose- and time-dependent manner by western blot analysis. As shown in Fig. 5c, with drug administration at a concentration of 6 µM for 0 h, 24 h, 48 h or 72 h, the expression of IKBKE, YAP1, TEAD2, Axl and c-myc gradually decreased over time; however, that of LATS2 and p-YAP1 (S127) progressively increased in U87-MG and LN229 cells, with an increase particularly noted at 72 h (Figure S3). Moreover, as shown in Fig. 5d, when increasing concentrations including 0 μM, 1 μM, 3 μM and 6 μM were administered for 72 h, the protein levels of IKBKE, YAP1, TEAD2, Axl and c-myc gradually decreased, while those of LATS2 and p-YAP1 (S127) increased, especially for the drug concentrations over 3 μM (Figure S3). However, it should be noted that the decrease in IKBKE expression resulting from CYT387 treatment was mainly due to posttranslational protein modification rather than effects at the transcriptional level, as IKBKE mRNA levels were determined to be negligibly changed by using real-time RT-PCR to evaluate dose- and time-dependent effects on U87-MG and LN229 cells (Additional file 1: Figure S1a, b). We next verified that CYT387 could reverse the inhibition of the Hippo pathway resulting from the overexpression of IKBKE. As shown in Fig. 5e, the expression levels of IKBKE, YAP1, TEAD2, Axl, and c-myc were increased with IKBKE overexpression and then decreased with 6 µM CYT387 treatment for 72 h. However, the expression trends for LATS-2 and p-YAP1 (S127) were contrary to those for YAP1 and TEAD2 (Figure S3). All of the above data demonstrated that CYT387 could enhance the activity of the Hippo pathway, which reduced YAP1, TEAD2 and downstream target protein expression, to inhibit glioblastoma progression by suppressing and inactivating IKBKE.

### IKBKE may directly interact with TEAD2 and YAP1 to regulate the Hippo pathway, accelerating TEAD2 and YAP1 translocation into the nucleus

**Fig. 6:**
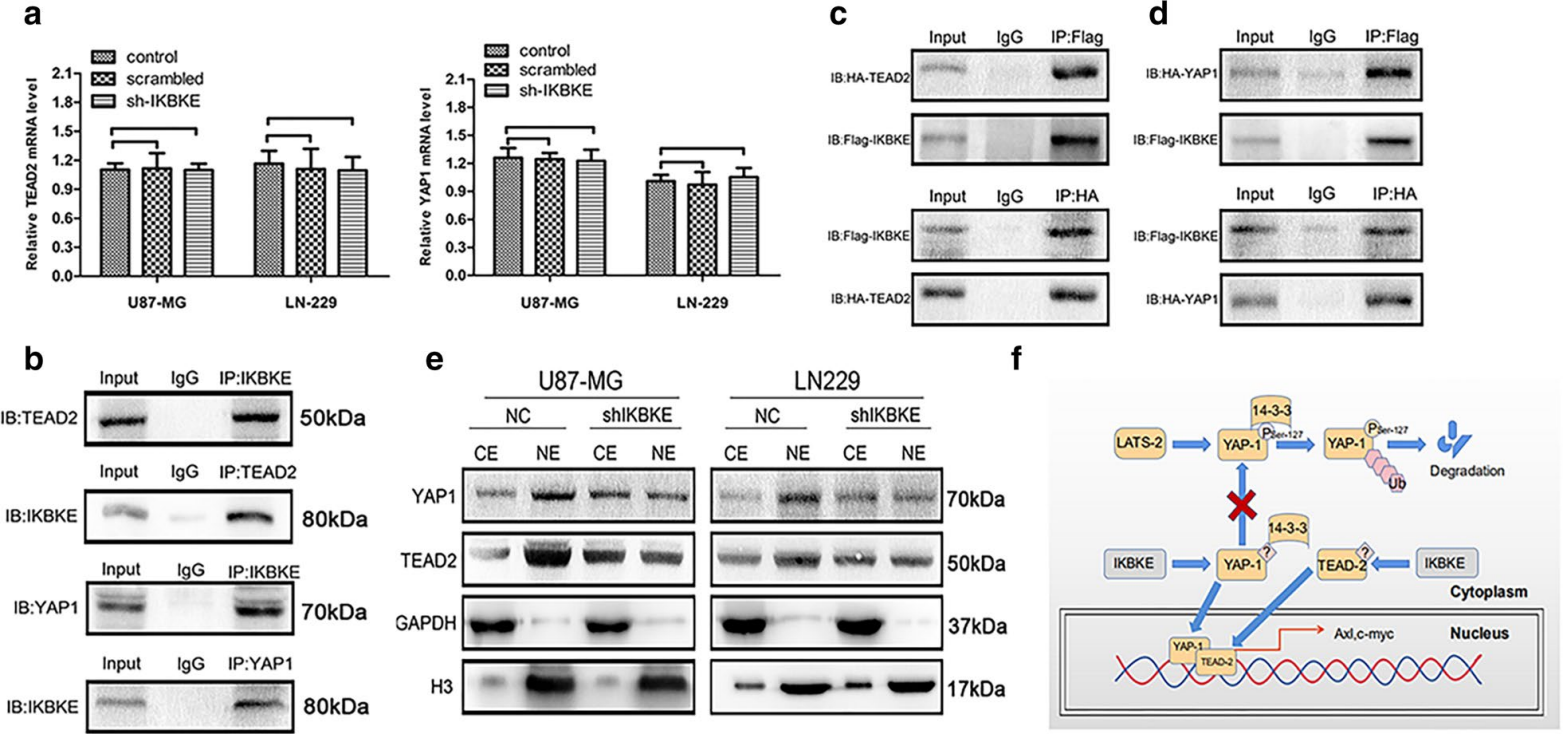
IKBKE directly interacts with TEAD2 and YAP1 to regulate the Hippo pathway, accelerating TEAD2 and YAP1 translocation to nucleus. **A** The mRNA expression of TEAD2 and YAP1 were analysized by real-time RT-PCR after knocking down IKBKE. **B** IKBKE directly interacted with TEAD2 and YAP1 using endogenous co-IP. **C** Flag-IKBKE combined with HA-TEAD2 using exogenous co-IP. **D** Flag-IKBKE combined with HA-YAP1 using exogenous co-IP. **E** Inhibition of IKBKE suppressed YAP1 and TEAD2 translocation to nucleus by western blot (CE, cytoplasmic extraction; NE, nuclear extraction). **F** Mechanism of IKBKE influencing on the Hippo pathway. All experiments were repeated at least three times

To investigate the detailed mechanism underlying the impact of IKBKE on the Hippo pathway, we first confirmed that IKBKE regulates YAP1 and TEAD2 at the posttranslational level. As shown in Fig. 6a, the mRNA expression of TEAD2 and YAP1 was hardly changed in the IKBKE-knockdown group compared to the blank control and scrambled groups, showing that inhibition of IKBKE decreased YAP1 and TEAD2 levels via posttranslational modification rather than changes in the mRNA levels. Next, we verified that IKBKE can directly interact with TEAD2 and YAP1 using endogenous co-IP (Fig. 6b and Figure S4). Then, we used Flag-IKBKE and HA-TEAD2 plasmids to perform exogenous co-IP. As shown in Fig. 6c, Flag-IKBKE could directly interact with HA-TEAD2 (Figure S4). We also performed exogenous co-IP using Flag-IKBKE and HA-YAP1 plasmids, showing that Flag-IKBKE interacted with HA-YAP1 (Fig. 6d and Figure S4). Additionally, we demonstrated that inhibition of IKBKE suppressed TEAD2 and YAP1 translocation into the nucleus using western blot analysis (Fig. 6e and Figure S4). All these data showed that IKBKE may directly interact with YAP1 and TEAD2 to promote YAP1 and TEAD2 transport into the nucleus. The detailed pathway diagram is shown in Fig. 6f.

### CYT387 inhibits tumor growth in a subcutaneous nude mouse model but has little impact on an intracranial orthotopic model

**Fig. 7:**
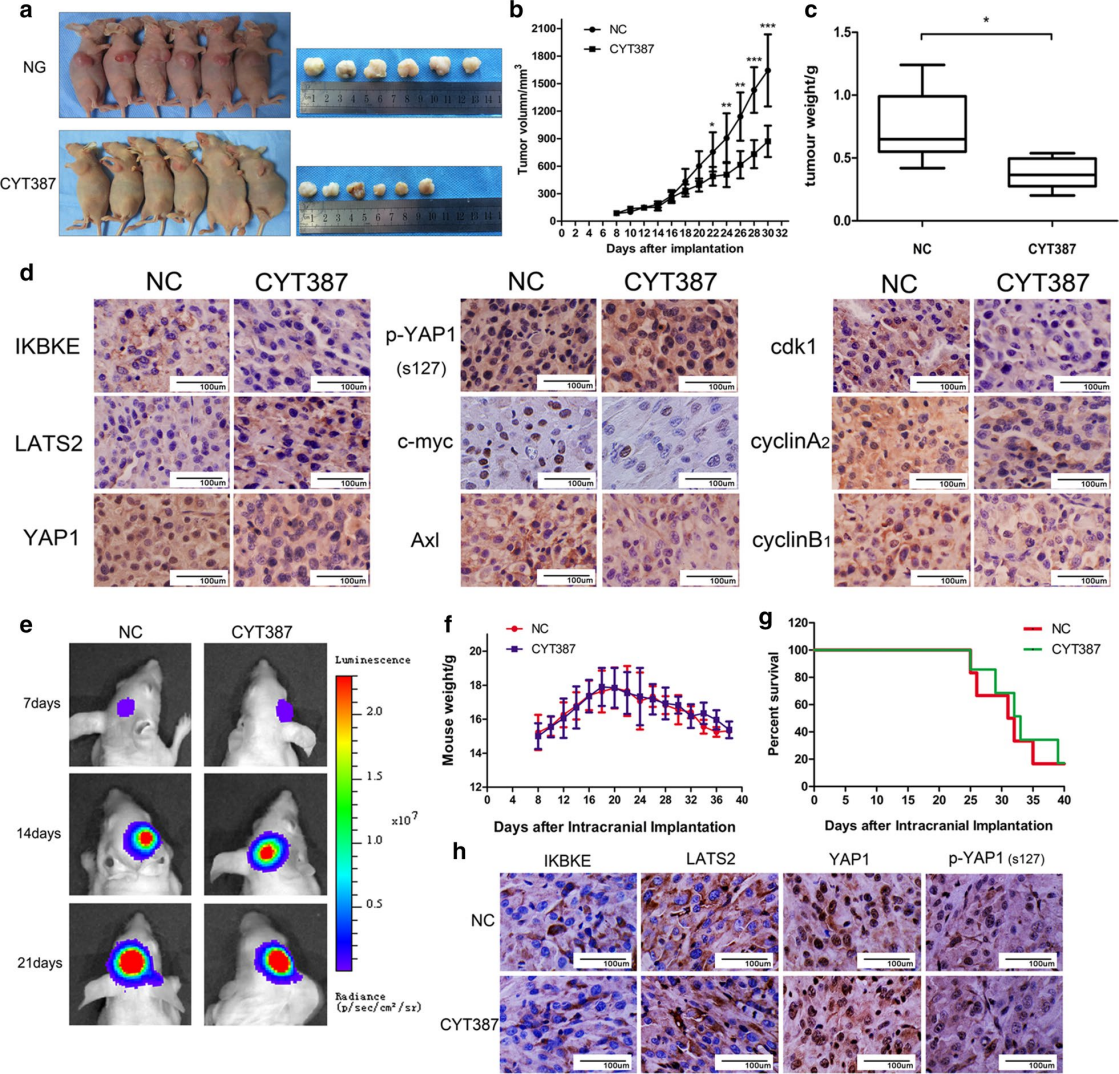
CYT387 inhibits subcutaneous tumour growth in nude mice but has little impact on intracranial orthotopic tumour. **A**, **B** Nude mouse subcutaneous tumor volume was monitored after fed with CYT387 (100 mg/kg/day). **C** Tumor weight was measured after fed with solvent or CYT387 for 30 days. **D** The expressions of IKBKE, LATS-2, YAP1, p-YAP1, Axl, c-myc, cdk1, cyclinA_2_ and cyclinB_1_ were detected by immunohistochemistry. **E** Intracranial tumors were monitored by luminescence imaging on 7th, 14th and 21th day after orthotopic xenotransplantation. **F** Mouse weight was monitored every 2 days after orthotopic xenotransplantation. **G** The survival rate was presented after nude mice fed with solvent or CYT387. **H** The expressions of IKBKE, LATS-2, YAP1 and p-YAP1 were measured by immunohistochemistry. All immunohistochemical stainings were repeated at least three times

To confirm whether CYT387 can inhibit tumor growth in vivo, we first established a subcutaneous nude mouse model via U87-MG cell inoculation. We used 6 mice fed the same concentration of solvent as the negative control (NC) group and 6 mice fed CYT387 at a dose of 100 mg/kg/day as the experimental group. Tumor volume was monitored every two days, and all mice were euthanized to measure implanted tumor weight on the 30th day. As time passed, the size of the tumors in the mice treated with CYT387 was obviously decreased (Fig. 7a, b) (P < 0.05), and the weight of these tumors was lower than that of the tumors in the NC group (Fig. 7c) (P < 0.05). Then, the expression of IKBKE, YAP1, Axl and c-myc was found to be downregulated, while that of LATS2 and p-YAP1 (S127) was shown to be increased in the tumors in the CYT387 (100 mg/kg/day) group compared with those in the NC group by immunohistochemical staining (Fig. 7d). Additionally, the levels of cell cycle markers such as Cdk1, CyclinA2 and CyclinB1 were reduced after mice were fed CYT387 compared with NC treatment (Fig. 7d). To explore whether CYT387 has an inhibitory effect on intracranial glioblastoma-like subcutaneously implanted tumors, we next established an orthotopic xenograft model with nude mice divided into two groups: an NC group fed the same concentration of solvent and a drug group fed CYT387 (100 mg/kg/day); U87-MG cells infected with a luciferase-expressing lentivirus were used. Imaging of intracranial tumor size was performed every 7 days after orthotopic xenotransplantation, and mouse weight was measured every 2 days. As shown in Fig. 7e, luminescence imaging showed no obvious differences between the NC group and the drug group at 7, 14, and 21 days. The weights and survival rates of the two groups of mice also showed few significant differences (Fig. 7f, g). Furthermore, we assessed IKBKE, LATS2, YAP1, and p-YAP1 (S127) expression levels by immunohistochemical staining, showing that there were few significant differences in these targets between the drug group and the NC group (Fig. 7h). These data suggested that CYT387 could inhibit glioblastoma progression in subcutaneous tumors but had little effect on intracranial orthotopic xenografts.

## Discussion

CYT387 (momelotinib), a JAK1/2 kinase inhibitor tested in clinical trials for myelofibrosis, has recently been identified as a potent IKBKE inhibitor. Zhu et al. [22] revealed that CYT387, which disrupts a cytokine circuit involving CCL5, IL-6, and STAT3, could suppress KRAS-dependent lung cancer cell growth. Barbie et al. [23] reported that CYT387 inhibited breast cancer proliferation via IKBKE, inducing NF-κB and STAT3 activation, while inhibition of JAK alone did not have the same inhibitory effect. JAK/STAT3 signaling has also been demonstrated to be central to GBM, and many small-molecule JAK inhibitors have produced positive results in in vitro and in vivo studies of GBM. In view of these findings, CYT387 may be a promising candidate inhibitor for gliomas due to its dual targeting. Here, we found that CYT387 decreased IKBKE expression levels in a dose- and time-dependent manner, especially with drug treatment for 72 h. However, this action may be dominated by posttranslational protein modification rather than changes at the transcriptional level, given that real-time RT-PCR analysis showed that IKBKE mRNA expression was negligibly altered by treatment with CYT387 (Additional file 1: Figure S1a, b). We also confirmed that CYT387 suppressed glioblastoma cell proliferation, migration, and invasion; promoted cell apoptosis; and induced cell cycle arrest at the G2/M checkpoint in vitro.

In this paper, we focused on exploring the novel mechanism by which CYT387, as a potent IKBKE inhibitor, inhibits the malignant progression of human glioblastoma. Recent studies have shown that IKBKE dominates cancer progression induced by the NF-κB pathway. For example, IKBKE phosphorylates CYLD and TRAF2 in breast cancer cells, which induces NF-κB activation and contributes to cell transformation [35, 36]. Guo et al. [37] also showed that IKBKE repressed FOXO3a primarily through direct phosphorylation of Ser644, which was found to promote cell survival, growth and tumorigenesis. In this paper, we proposed that IKBKE can regulate the Hippo pathway by directly interacting with YAP1 and TEAD2 and promoting YAP1 and TEAD2 transport into the nucleus. Previous data indicated that the Hippo signaling pathway might contribute to glioblastoma progression. Ji et al. [26] reported that LATS1 expression is significantly downregulated in glioma; furthermore, reduced LATS1 expression is markedly negatively correlated with the WHO grade and overall survival time. Orr et al. [27] demonstrated that elevated nuclear immunoreactivity of YAP1 was prominent in high-grade gliomas, suggesting the potential role of YAP1 in the pathobiology of the most common malignant brain tumors. Therefore, we speculated that IKBKE may promote glioblastoma progression via regulation of the Hippo pathway.

Our studies also showed that CYT387 decreased YAP1 and TEAD2 expression and increased LATS-2 and p-YAP1 (S127) expression to strengthen Hippo pathway activity mainly induced by inactivation of IKBKE. We also found that IKBKE knockdown enhanced Hippo pathway activity predominantly through direct interactions with YAP1 and TEAD2, which inhibited YAP1 and TEAD2 transport into the nucleus. Recently, Lue et al. [20] showed that CYT387 inhibited total YAP1 expression and enhanced p-YAP1 (S127) expression in renal cell carcinoma cell lines, and we discovered, for the first time, that this function was mainly induced by inhibition of IKBKE. LATS1/2 expression can be dominated by YAP expression via a negative feedback loop in the Hippo pathway [38, 39].

Recent studies have reported that caspase-9 triggers cell apoptosis following cleavage and activation after being sensitized by apoptosomes [40, 41]. We also verified by western blot analysis that the antiapoptotic protein Bcl-2 and total caspase-9 levels were decreased, while those of proapoptotic Bax and cleaved caspase-9 were increased by drug treatment, demonstrating that CYT387 could promote glioblastoma cell apoptosis. Furthermore, we showed that CYT387 induced cell cycle arrest at the G2/M checkpoint. According to previous studies, CyclinA2 participates in the regulation of the S phase as well as mitotic entry and is also a marker of cell proliferation and invasion [42]. The CyclinB1-Cdk1 complex, a key regulator of mitotic entry, dominates mitosis skipping, arresting cells at the G2/M checkpoint via inactivation of Cdk1 kinase by degradation of cyclinB1 [43]. Cdc25C can fully activate CyclinB1-Cdk1 after translocation into the nucleus by directly dephosphorylating Cdk1 to induce G2/M progression [44, 45]. Through western blot analysis, CyclinA2, CyclinB1, Cdk1 and Cdc25c expression was found to be obviously decreased after drug treatment, while the expression of CyclinD1, an essential factor that regulates the cell cycle transition from the G1 to S phase [46], was negligibly altered, suggesting that cells treated with CYT387 undergo cell cycle arrest at the G2/M checkpoint. In previous investigations, similar results were obtained, showing that increased LATS1 expression inhibited cell proliferation by blocking the G2/M transition, mainly through inhibition of the kinase activity of the Cdc2/Cyclin A/B complex [47].

In a following experiment, we verified that CYT387 could inhibit tumor growth in a subcutaneous nude mouse model but found that CYT387 had little effect on an intracranial orthotopic model. The expression of IKBKE, YAP1 and Hippo downstream factors such as c-myc and Axl was found to be decreased, while the expression of LATS-2 and p-YAP (S127) was shown to be increased in the drug group using immunohistochemical staining, indicating Hippo pathway activity in subcutaneous tumors was enhanced after drug treatment. However, no obvious changes in IKBKE or Hippo activity were observed in the intracranial xenografts. We hypothesized that this was probably due to the limited ability of CYT387 to penetrate the blood–brain barrier (BBB). According to previous research, Durmus et al. [48] reported that the cerebral concentration of CYT387 was far lower than that in the plasma but that it was dramatically increased in Bcrp1−/−; Mdr1a/1b−/− mice compared with WT mice, while the plasma concentration was little impacted, demonstrating that cerebral accumulation of CYT387 is likely restricted by Mdr1a/1b and Bcrp1. Therefore, it is possible that CYT387 does not reach an effective concentration in the brain to suppress intracranial glioblastoma progression, and the ability of this drug to pass through the blood–brain barrier (BBB) urgently needs to be improved. A recent study showed that lipid-core nanocapsules act as drug shuttles through the BBB, delivering drugs to the brain tissue with high efficiency and reducing glioblastoma after intravenous or oral administration [49]. Thus, in the future, CYT387 will be encapsulated in nanocapsules for shuttling across the BBB to enable it to exert an antitumor effect.

## Supplementary Information

**Additional file 1: Figure S1.** CYT387 hardly changes IKBKE mRNA expression in a dose-dependent and a time-dependent manner. **Figure S2.** Original western blots used in the Figs. 2, 3 and 4. **Figure S3.** Original western blots used in the Fig. 5. **Figure S4.** Original western blots used in the Fig. 6.

## Data Availability

The data that support the findings of this study are available from the corresponding author upon reasonable request. And all WB bands have been added to Additional file 1: Figure S1.
