# Supplementary material for: CYT387, a potent IKBKE inhibitor, suppresses human glioblastoma progression by activating the Hippo pathway
Source: J Transl Med. 2021 Sep 20;19:396. doi: 10.1186/s12967-021-03070-3 (PMC8454155; doi:10.1186/s12967-021-03070-3)

**Additional Information**

**Figure S1.**

CYT387 hardly changes IKBKE mRNA expression in a dose-dependent and a time-dependent manner.

**Figure S2.**

Original western blots used in the figure 2, figure3 and figure4.

**Figure S3.**

Original western blots used in the figure 5.

**Figure S4.**

Original western blots used in the figure 6.


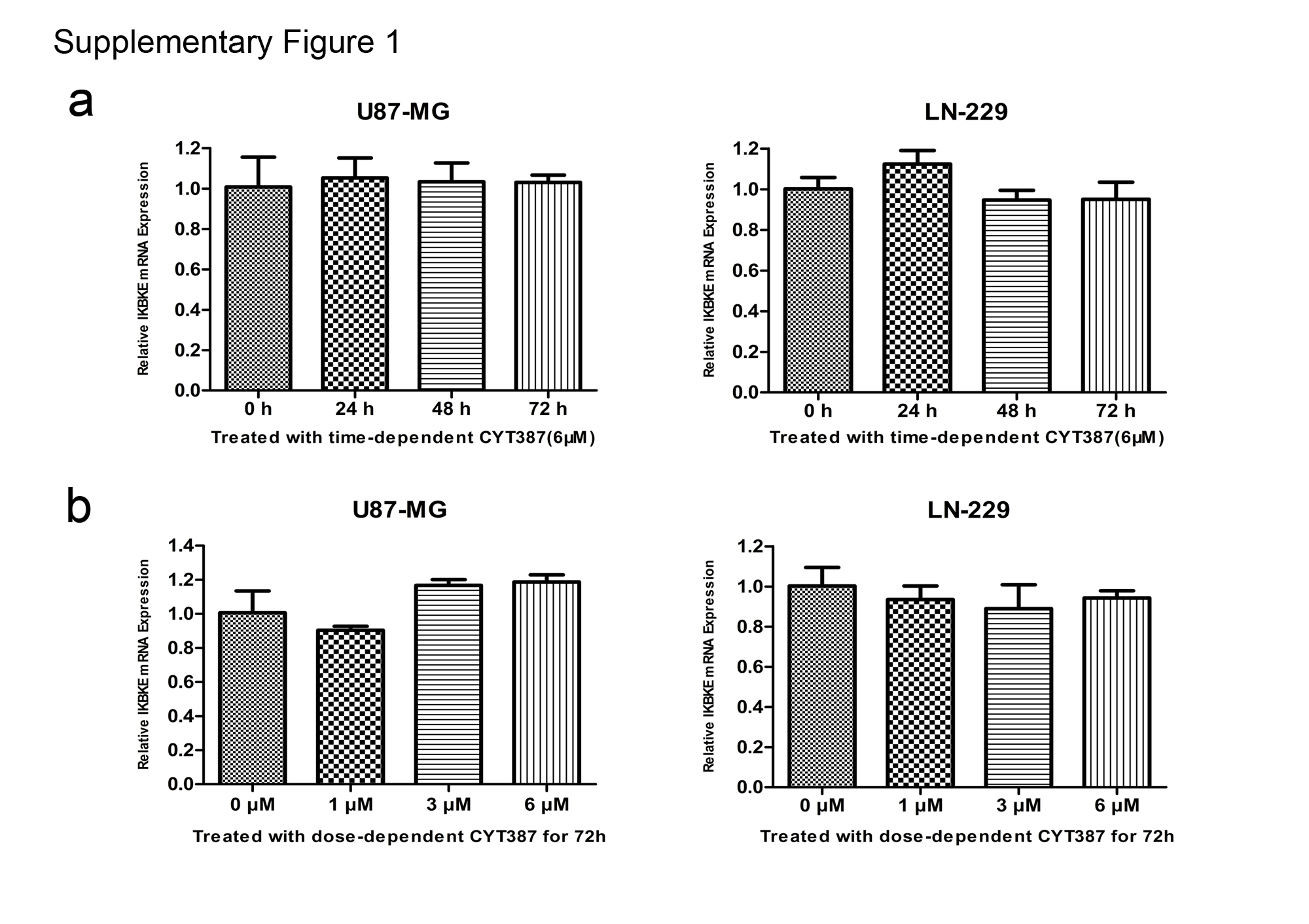


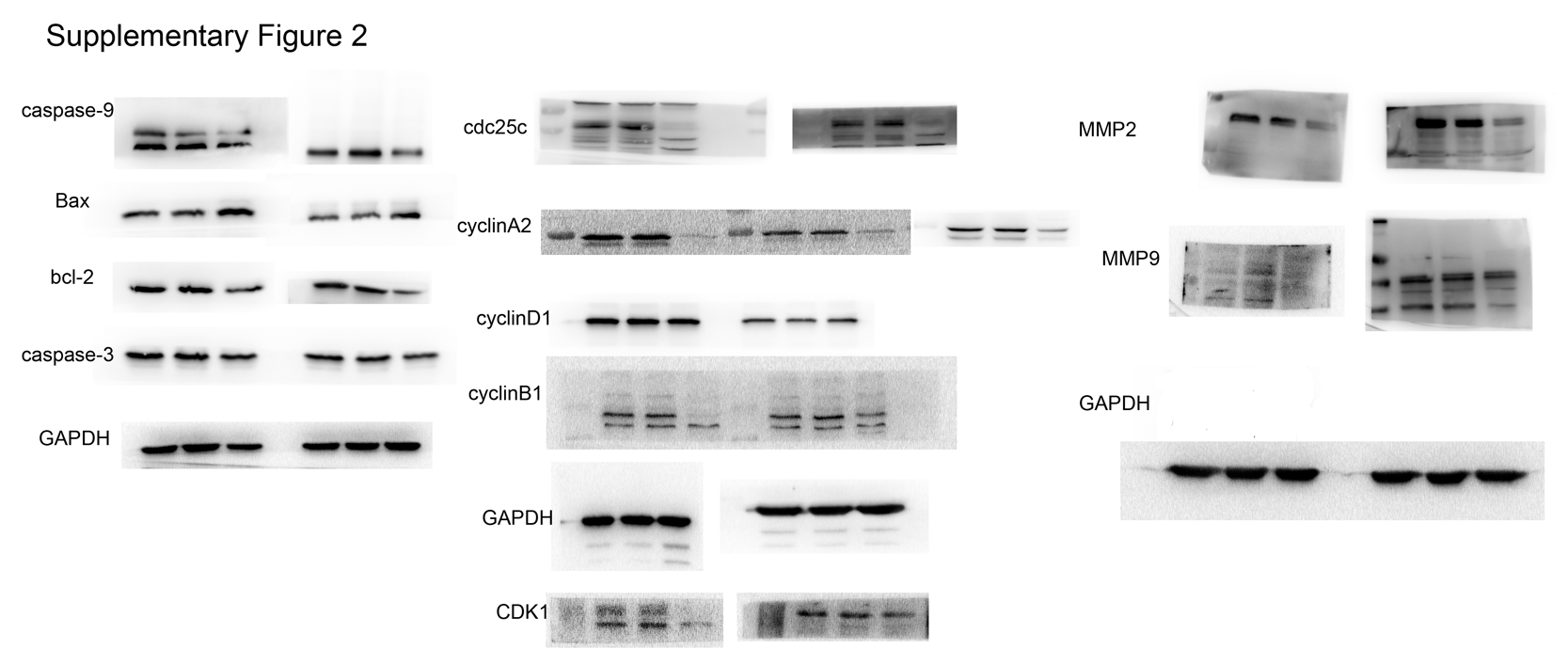


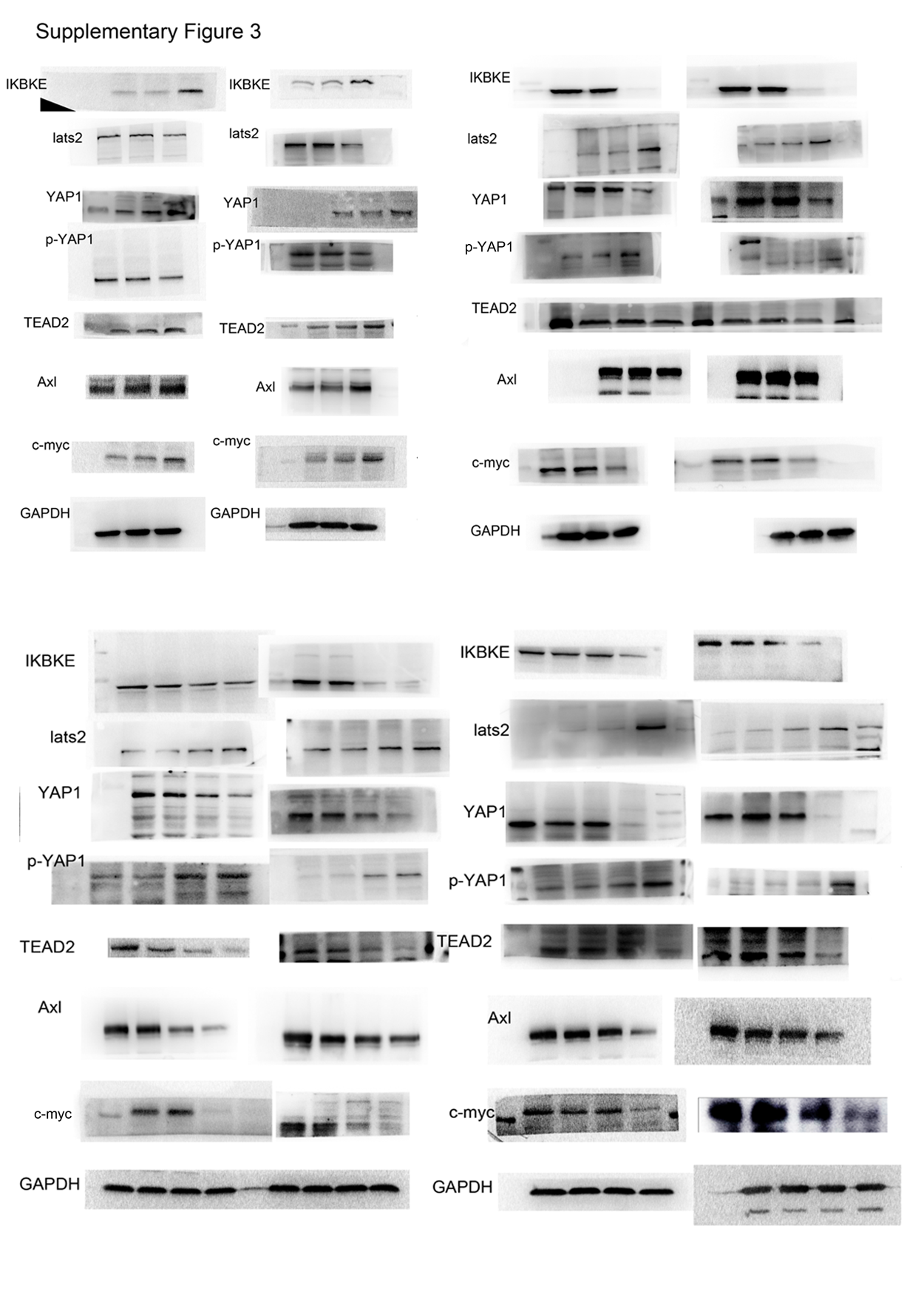


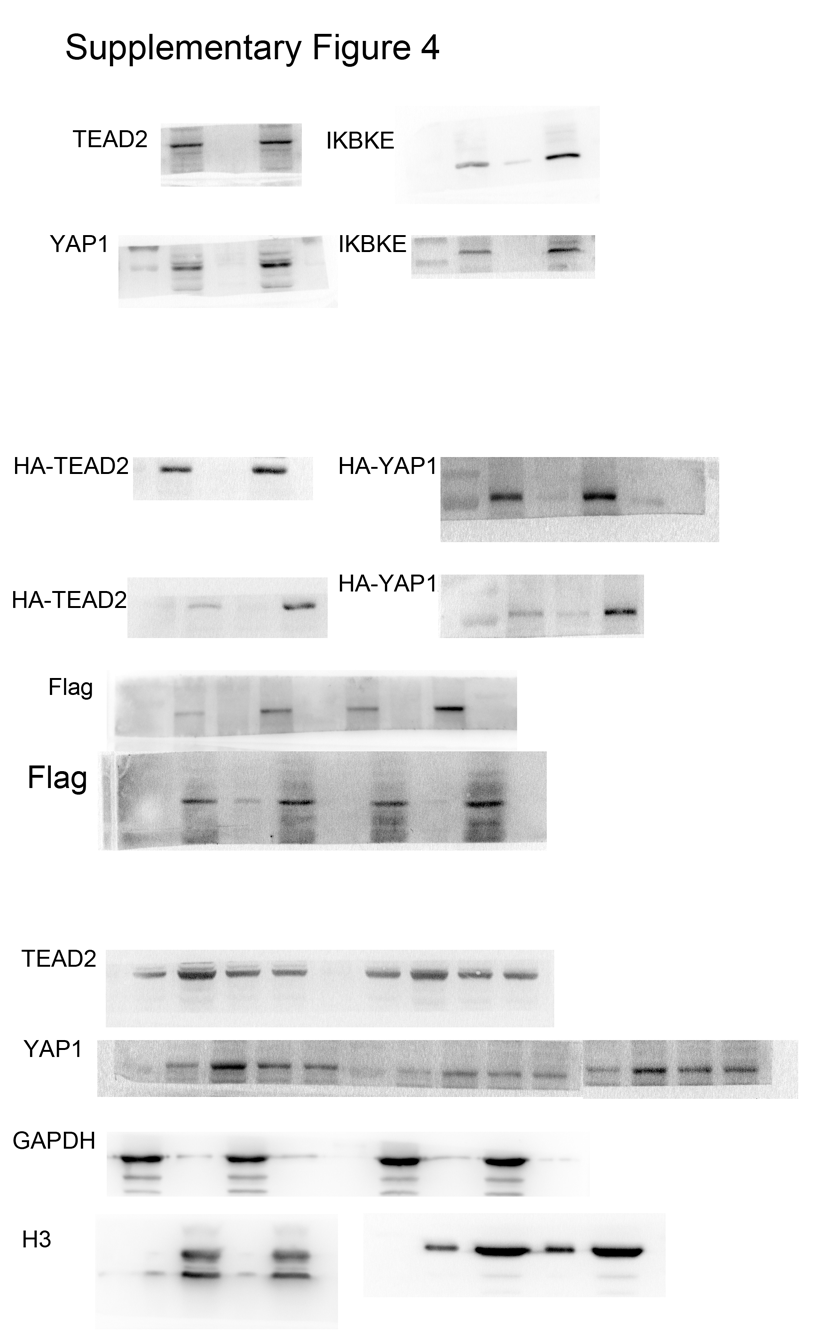

Supplement: Supplementary file 1 — Additional file 1: Figure S1. CYT387 hardly changes IKBKE mRNA expression in a dose-dependent and a time-dependent manner. Figure S2. Original western blots used in the Figs. 2, 3 and 4. Figure S3. Original western blots used in the Fig. 5. Figure S4. Original western blots used in the Fig. 6. [file 12967_2021_3070_MOESM1_ESM.doc]
